# Application of Medical Image Navigation Technology in Minimally Invasive Puncture Robot

**DOI:** 10.3390/s23167196

**Published:** 2023-08-16

**Authors:** Shuai Hu, Rongjian Lu, Yinlong Zhu, Wenhan Zhu, Hongzhe Jiang, Suzhao Bi

**Affiliations:** School of Mechanical and Electronic Engineering, Nanjing Forestry University, Nanjing 210037, China; hushuai@njfu.edu.cn (S.H.);

**Keywords:** microneedle puncture, puncture robot, path planning, image navigation, surgical-efficacy testing

## Abstract

Microneedle puncture is a standard minimally invasive treatment and surgical method, which is widely used in extracting blood, tissues, and their secretions for pathological examination, needle-puncture-directed drug therapy, local anaesthesia, microwave ablation needle therapy, radiotherapy, and other procedures. The use of robots for microneedle puncture has become a worldwide research hotspot, and medical imaging navigation technology plays an essential role in preoperative robotic puncture path planning, intraoperative assisted puncture, and surgical efficacy detection. This paper introduces medical imaging technology and minimally invasive puncture robots, reviews the current status of research on the application of medical imaging navigation technology in minimally invasive puncture robots, and points out its future development trends and challenges.

## 1. Introduction

Currently, less invasive and minimally invasive treatment has become the primary trend in the development of modern clinical medicine. Compared with traditional surgical treatment methods, minimally invasive surgical treatment has the advantages of minor wounds in the lesion area, less pain, faster postoperative recovery, shorter hospital stay, and less bleeding [1,2]. Microneedle puncture is widely used in medical diagnosis and surgery, and typical applications include microneedle puncture to extract blood or tissue and its secretions for pathological examination, targeted drug delivery, local anaesthesia, microwave ablation therapy [3,4,5,6,7], etc.

With the development of modern industrial technology and the continuous improvement of clinical medicine, researchers have applied robots in minimally invasive puncture surgery, improving the accuracy, efficiency, and safety of minimally invasive puncture surgery. Medical image navigation technology has a wide range of applications in minimally invasive puncture robots, mainly including the following aspects: (1) image acquisition: minimally invasive puncture robots can be equipped with different types of image acquisition equipment (e.g., Ultrasound (US), Computed Tomography (CT), magnetic resonance imaging (MRI) [8], etc.) to obtain the corresponding image data; (2) image processing: the acquired image data are processed as needed to obtain clear and accurate image data; (3) image navigation: the image data are fused with the control system of the robot to achieve real-time navigation and positioning [9]; (4) automated puncture [10]: some minimally invasive puncture robots can automate puncture and automatically locate the puncture point and depth of punch through image navigation technology, reducing the difficulty and risk of the surgeon’s operation. In conclusion, medical imaging navigation technology has made it safer and more efficient for robots to perform minimally invasive puncture surgery.

In 2020, the global market size of percutaneous puncture surgery robots will be 380 million US dollars, of which the Chinese market will account for 5.48%. From 2018 to 2020, the growth rate of China’s percutaneous puncture surgery robot market is maintained at about 37–39%, accounting for 4.89% of the overall Chinese surgical robot market, which is the third largest surgical robot market segment except for endoscopic surgery robots and orthopaedic surgery robots [11]. Although minimally invasive puncture robot technology has obvious advantages and broad market prospects, there are still some technical and practical challenges in actual clinical application.

## 2. Overview of Medical Imaging Technology

### 2.1. Definition of Medical Image Navigation Technology

Medical image navigation technology is a technology that combines medical imaging and clinical medicine field operations. Common medical navigation techniques include the following: ultrasound, CT, MRI, optical tracking, endoscope and so on [12]. By processing and analysing real-time medical images, doctors generate corresponding two-dimensional or three-dimensional images and combine them with the actual anatomy of the patient so that they can precisely determine the location and direction of the operation and improve the efficiency, accuracy, and safety of the procedure [13,14,15].

Medical imaging navigation technology is widely used in tumour treatment, interventional surgery, minimally invasive surgery, and other medical fields. Medical imaging provides comprehensive, precise, and efficient imaging of the human body in a non-invasive or minimally invasive way, providing objective and reliable diagnostic information to help doctors develop the best treatment plan.

### 2.2. Classification of Medical Image Navigation Technology

#### 2.2.1. Single-Modal Medical Imaging

Single-modal medical imaging techniques refer to methods that use a single type of medical imaging technology to acquire medical image information. Common Single-modal imaging techniques include ultrasound, CT, MRI [16,17,18], etc. Single-modal medical imaging techniques have been widely used in medical imaging and clinical practice, and the technology provides information about pathology, anatomy, and physiology that is important for detecting, diagnosing, and treating disease [19].

Different models of medical imaging technology have various advantages and limitations. Ultrasonic imaging is radiation free, simple to operate, provides real-time results, and is low cost [20]. CT imaging is fast and widely used to diagnose diseases of the skeletal system, chest, abdomen [21,22], etc. The imaging process of MRI is radiation free and non-invasive to the human body, and the imaging effect on soft tissues, bone marrow, joints, and other parts is better than CT [23,24].

A single type of medical imaging information cannot provide comprehensive physiological and anatomical information [25]. Due to various factors, single-modality medical imaging technology has limitations [26]. The penetration ability of ultrasound is limited, and sometimes it is challenging to obtain image information of tissues and organs with greater depth or complex structures. The imaging effect is poor [27]. CT imaging produces radiation and is easily affected by the patient’s position and respiratory movements, making it difficult to differentiate between tissues and organs with similar tissue density [28,29]. MRI imaging time is extended, limited by the patient’s body position, breathing, and other factors, which may affect the quality and accuracy of imaging, and susceptible to interference from metallic substances, oxidized particles [30,31,32], etc.

#### 2.2.2. Multimodal Medical Imaging

Currently, medical imaging has evolved from single-modal to multimodal. Multimodal medical image fusion technology refers to integrating and registering different medical imaging information (such as ultrasound, CT, MRI, PET, etc.) to obtain more accurate, reliable, and real-time medical imaging technology than single-modal images [17,33]. Multimodal medical photos make up for the defects of single-modal medical imaging, and the fused medical images retain the characteristic information of the original pictures with more affluent and comprehensive information [34].

Common multimodal medical imaging techniques include PET-CT (Positron Emission Tomography–Computed Tomography), SPECT (Single Photon Emission Computed Tomography), PET-MRI (Positron Emission Tomography/magnetic resonance imaging), CT-MRI [35,36], etc. Multimodal imaging techniques have the following advantages: (1) Comprehensive information: Combining multiple imaging techniques allows for more comprehensive and accurate information. For example, MRI can provide detailed information on tissue structure, while PET can provide metabolic information. PET-MRI can see the structure and information of tissues simultaneously, giving a more comprehensive understanding of the lesion [37,38]. (2) Improve diagnostic correctness: multimodal imaging technology can improve the correct disease diagnosis rate and reduce the risk of missed and misdiagnosis. PET-CT imaging can help differentiate between benign and malignant tumours, improving diagnostic accuracy [38,39]. (3) Radiation dose reduction: The use of multimodal imaging can reduce the radiation dose received by patients [40]. (4) Improve surgical outcomes: Multimodal imaging can help doctors better plan surgical plans and improve surgical outcomes [41].

### 2.3. Application of Medical Image Navigation

Medical image navigation technology is a technique that precisely matches real-time image information to the patient’s body and is widely used in various surgical and therapeutic procedures. Its main application areas are shown in Table 1.

With the development and advancement of technology, medical image navigation technology has also extended many expanded application areas, bringing more benefits to medicine and patients, for example, combined with technologies such as robotic surgery systems, augmented reality (AR), and virtual reality (VR) [52,53]. This is expected to increase the procedure’s precision and safety further and improve the patient’s treatment experience.

## 3. Overview of Minimally Invasive Puncture Robot

### 3.1. Definition of Minimally Invasive Puncture Robot

Minimally invasive puncture robot is a robotic system that performs high-precision, highly flexible puncture procedures through a robotic arm and its control system [54,55]. It enables the puncture of tissues, organs, or other body parts inside the patient’s body through precise control and navigation for treatment or examination [56,57,58]. A complete minimally invasive puncture robot system generally includes a robotic arm, imaging system, puncture needle and catheter, control system, human–machine interface [59,60,61,62,63,64], etc.

### 3.2. Advantages of the Minimally Invasive Puncture Robot

Robot-assisted minimally invasive surgery is a new field and has become a research hotspot in medicine and engineering. The specific advantages and disadvantages of minimally invasive puncture robots can be seen from Table 2:

Through precise control, advanced sensor technology and stable operation, the robot can eliminate the influence of human factors such as hand shaking, ensuring accurate operation. In interventional radiation therapy or puncture surgery that requires the support of radiological imaging equipment such as CT, the use of robotic puncture can reduce the radiation dose received by the medical staff. Through virtual reality and simulation training, it is easier for doctors to master minimally invasive puncture skills and improve the overall surgical level. Procedures can be standardised, helping to ensure consistency across hospitals and between doctors, thereby improving treatment outcomes.

Minimally invasive puncture robots can provide more precise and personalized surgical programs to meet the needs of different patients, which will continue to promote the development of robot technology, but also promote the innovative integration of artificial intelligence, the Internet of things, biomedical engineering and other disciplines. With the growth of global medical demand, minimally invasive puncture robots are expected to find broader market opportunities worldwide.

The research and development, purchase, and installation and maintenance costs of minimally invasive puncture robots are relatively high, and the operation of minimally invasive puncture robots requires certain professional skills and training, which may increase the learning pressure and training costs of medical staff. Compared with traditional surgical methods, minimally invasive puncture robots may have problems in man–machine communication and coordination, which may affect the smooth operation of the surgical process.

Robotic systems can encounter malfunction or failure, and any technical malfunction can cause serious harm to the patient. Some patients and medical staff may have doubts and concerns about the new technology, and their trust in robotic surgery may be low, which may limit the acceptance and adoption of robotic puncture technology. Relevant laws and regulations will also limit the promotion of robots. Robotic surgery may involve sensitive patient data, and without appropriate security measures, there may be a risk of data breaches and privacy violations.

### 3.3. Research Progress of Minimally Invasive Puncture Robot

Robots have been used in the medical field for 30 years. In 1985, the first robot for surgery, PUMA 200, was born [65]. Since then, research on surgical robots has been increasing. In recent years, significant progress has been made in the study of minimally invasive puncture robots. The control method can be divided into three categories: manually controlled, semi-automatically controlled, and automatically controlled.

Manual control means the physician directly manipulates the robot’s movement through a remote control, computer interface, or other operating devices to achieve precise puncture operations. Zhou et al. [66] developed a new method of using a surgically guided puncture robotic system to locate small lung nodules before thoracoscopic surgery, in which the robot is used for positioning and then manually punctured. Manual control can give full play to the physician’s expertise and respond flexibly to clinical situations. Still, the manual operation process may be influenced by human factors, resulting in limited operational accuracy and stability.

In response to these problems, several research institutions have conducted extensive studies to improve the accuracy of robotic punctures and shorten puncture time. Compared with manual control, the semi-automatic control of the puncture robot has higher accuracy and stability, which can reduce the human factor in manual operation and improve the efficiency and safety of the procedure. At the same time, semi-automatic control will not completely replace the manual operation of doctors, who will still adjust the puncture based on the patient’s specific condition.

Siegfarth et al. [67] designed a semi-automated robotic system for percutaneous puncture that uses a hybrid system of manual and machine-automated operation that allows the physician to perform precise punches guided by CT. NDR Medical Technology [68,69] has launched a commercially available body-fixed puncture robot system called ANT-X. This system utilizes real-time fluoroscopy CT to adjust the puncture needle’s position and is manually punctured by a doctor. Hiraki et al. [70] developed a semi-automatic real-time puncture robot based on CT fluoroscopic navigation. This robot can achieve autonomous localization of the puncture needle, remote operation of puncture, and has conducted experiments on animals and human bodies. The overall needle placement error is around 1.6 mm.

Based on the semi-automatic control puncture robot, the automatic control puncture robot is more automated and intelligent. Automatic control of the puncture robot can realize automatic control and adjustment of the needle and complete the puncture operation autonomously. Hou et al. [71] designed an automated robotic puncture system based on optical surgical navigation for the precise ablation of liver cancer. Yang et al. [72] developed a puncture robot for fast and accurate lung biopsy. The robot incorporates a four-degree-of-freedom positioning module for locating the puncture point, which is suitable for most positions on the patient’s chest.

Automatic control puncture robots have higher accuracy and efficiency, avoiding human factors in manual operations, reducing the workload and operational risks for doctors, and improving surgeries’ success rate and safety. Although the intelligence and automation level of automated control puncture robots is higher, they still require the supervision and operation of doctors to ensure the success and safety of surgeries. At the same time, further research and exploration are needed to apply automated control puncture robots to meet clinical practice needs.

All in all, these three types of minimally invasive piercing robots represent different levels from human to fully automated, and each type has its own unique advantages and challenges. Manual control of the piercing robot provides maximum operational flexibility, and the doctor can control and adjust the robot’s movements in real time, but the doctor’s skills and experience are demanding, increasing the complexity of the operation and the risk of error. Semi-automatic control can improve the accuracy and consistency of the operation, while still allowing the doctor to maintain a degree of control, but requires precise coordination and monitoring of the robot and the operation process, adding to the complexity of the system. Automatic control maximizes the accuracy and repeatability of the operation and reduces the work burden of the doctor, but the accuracy and reliability of the robot are very high, and once there is an error or failure, it may be difficult to intervene in time, and there are safety and compliance risks.

## 4. Current Status of Research on the Application of Medical Image Navigation Technology in Puncture Robots

### 4.1. Overview of Research on the Application of Medical Image Navigation Technology in Puncture Robots

Medical image navigation technology provides real-time navigation guidance for the puncture robot, and by processing and analysing medical image data, it can achieve precise positioning of the puncture target, as well as provide feedback and adjustment based on real-time image data during the puncture process to ensure the accuracy and safety of the puncture [73,74,75,76].

Medical image navigation technology has been widely used in puncture robots, including tumour puncture, nerve puncture, interventional procedures, and many other fields. The application of medical image navigation technology improves the accuracy and safety of surgery, shortens the operation time, reduces injury to patients, and is significant in enhancing the level and quality of medical care.

### 4.2. Case Study of Medical Image Navigation Technology in a Puncture Robot

#### 4.2.1. Ultrasound-Technology-Guided Robotic Puncture

Ultrasound imaging is non-invasive, convenient, and intuitive medical imaging method with good real-time imaging and low imaging cost. Zhang et al. [77] designed a visualization system for automatic ultrasonic scanning and surgical path planning for lumbar puncture, which can realize three-dimensional reconstruction visualization and full-dimensional stereoscopic display, providing image reference for puncture surgery. Lin et al. [78] proposed an ultrasound-guided precise percutaneous puncture robot system. Under the guidance of ultrasonic images, the average puncture error of the robot puncture was 1.09 ± 0.35 mm. Since the difference in tissue deformation between scanning and puncture procedures cannot be completely eliminated, the system still requires manual fine-tuning of the puncture path. Chen et al. [79] implemented a coordinate transformation between the robot and the ultrasound image utilizing an ultrasound probe mounted inside the puncture robot and using a novel calibration method. The system allows the operator to plan the puncture target and the puncture path on the ultrasound image, and the robot automatically performs the needle insertion. With the assistance of the robotic system, the positioning and orientation accuracy of the needle insertion were 0.9 ± 0.29 mm and 0.76 ± 0.34°, respectively. The robotic system cannot handle anatomical movements of the target caused by body movements (breathing or other bodily movements), and the patient needs to remain still and hold his breath for a while during needle insertion.

Ultrasound imaging provides a real-time visual guidance method for robotic puncture, allowing the physician to observe and monitor in real time during the procedure. In addition, it has the advantages of low cost, easy operation and wide application. However, the resolution and contrast of ultrasound imaging may not be as good as those of some other imaging techniques (e.g., CT or MRI). For some deep or complex anatomical structures, ultrasound may not provide adequate image quality.

#### 4.2.2. CT-Guided Minimally Invasive Puncture Robotic Puncture

CT-guided minimally invasive robotics is a method that uses computed tomography to guide a robotic puncture for tissue sampling or treatment. Zhang et al. [80] proposed a CT-guided robot-assisted coordinate-positioning puncture method. By performing experiments in rabbit livers, CT-guided robotic puncture can achieve comparable accuracy to manual puncture under the same conditions but with fewer CT scans and a lower radiation dose during robotic puncture.

Guo et al. [81] designed a four-degree-of-freedom puncture robot for CT-guided percutaneous transluminal biopsy. This robot improves the puncture accuracy of the developed robotic system through model-predictive control. Groetz et al. [82] performed puncture experiments with a robot mounted on a standard table of a CT scanner. In all cases, the biopsy needle can be accurately placed in the centre of the puncture target. The accuracy of the robotic puncture system in thoracoabdominal procedures will be compromised due to the lack of effective monitoring of respiratory movements.

Compared with the penetration ability of ultrasound, CT-guided robotic puncture is more suitable for deep tissue puncture, such as cancer treatment, liver, pancreas and lung surgery, and the imaging effect of CT is more significant. The CT-guided puncture robot combined with high-resolution three-dimensional imaging technology can improve the success rate of deep puncture. However, this technique also has some disadvantages, such as the risk of radiation exposure for patients and medical staff, high cost, limitations of operating space, and limitations of real-time feedback, which limit CT-guided robotic puncture in some cases.

#### 4.2.3. MRI-Guided Minimally Invasive Puncture Robotic Puncture

Magnetic resonance imaging (MRI) is primarily used for disease diagnosis, lesion assessment, monitoring the effectiveness of treatment, and conducting neuroscience research. Its advantage lies in the high-resolution imaging of soft tissues, which is especially suitable for examining the brain, spinal cord, joints, muscles, and other body parts [83,84,85,86]. From neurosurgery to percutaneous puncture, MRI-guided robot-assisted techniques have been widely studied and applied.

Fischer et al. [87] developed an MRI-compatible pneumatic robot for transconjunctival prostate needle placement. Krieger et al. [88] designed an MRI-guided transrectal prostate puncture machine. The robot uses a piezoelectric ceramic motor drive for needle guidance positioning and manual puncture. Franco and others [89] designed a needle robot for laser ablation (LA) of liver tumours guided by magnetic resonance imaging (MRI). The robot is provided within the MRI scanner bore for puncture needle alignment, and manual needle insertion is used. Lim et al. [90] developed a magnetic resonance imaging (MRI)-guided bone biopsy robot for paediatric patients. The robot is made entirely of non-metallic parts with pneumatic actuators and optical encoders. A visual servo-controlled robotic system for MRI-guided breast intervention was proposed by Zhang et al. [91]. This establishes a kinematic model of the robot and proposes a series–parallel robot control method that combines forward kinematics with visual servo control to achieve accurate needle control. The above specific comparisons are presented in Table 3.

The MRI-guided puncture robot combines high-resolution imaging capabilities with excellent contrast to soft tissue to provide a radiation-free solution for precise and complex puncture tasks. However, the high cost of this technology, operating space constraints, interaction with metal parts, delays in image updates and compatibility issues may limit its applicability in a particular environment or application.

#### 4.2.4. Multimodal Imaging Technology Guides Minimally Invasive Puncture Robot Puncture

In robot-assisted puncture, data acquisition and pre-processing, such as denoising and contrast enhancement, are first carried out for multimodal images. Then, the image fusion and registration are carried out, and the pictures of different modes are fused to obtain accurate anatomical structure information. Finally, target structures such as tumours and blood vessels are identified and extracted. On this basis, path planning is carried out to provide the optimal puncture path for the robot.

Balter et al. [10] developed a seven-degree-of-freedom (DOF) robotic system that combines near-infrared and ultrasound imaging for performing venipuncture. The robot consists of a three-degree-of-freedom gantry for imaging the peripheral veins of the patient’s forearm and a miniaturized four-degree-of-freedom serial arm for guiding the catheter into the selected vein under closed-loop control. Experimental results show that the puncture accuracy can reach a sub-millimetre level in the whole operating workspace of the manipulator, and the success rate of puncture in the skin simulation tissue model is high. Regarding the issue of CT being unable to provide real-time guidance, Shahriari et al. [92] designed a needle-steering puncture robot that integrates CT imaging and electromagnetic sensor data—tracking the puncture of a penetration robot using real-time electromagnetic sensor data to enhance precision and real-time performance.

In addition to ultrasound, CT, and MRI techniques to guide the robot for a puncture, Jiang et al. [93] studied a robot-assisted puncture system based on optical positioning technology. The robot uses binocular cameras for image acquisition, uses least squares to detect circles to identify circular markers, uses binocular-phase visual-depth information to solve for the coordinate information of the markers in 3D space, and then guides the robot to perform punctures. He et al. [94] designed a venipuncture robot with decoupled position and attitude guided by near-infrared vision and force feedback. It uses near-infrared images and laser sensors to obtain the three-dimensional information of puncture positions, while the change in force detects the state feedback of punctures. Guo et al. [95] designed a compact robotic guidance system that could accurately realize the needle position and orientation within the operating room. The eye-gaze tracking-based approach is proposed to control the status and direction of the needle toward the desired location in a more intuitive manner.

Targeted robot puncture guided by multimodal imaging technology combines near-infrared, ultrasound, CT, MRI, optical positioning and other imaging. Research shows that the sensitivity and accuracy of robot targeted puncture guided by multimodal imaging technology are higher than that of robot targeted puncture guided by a single image technology. However, the implementation and application of multimodal imaging technology has a certain complexity, and the data formats and characteristics generated by different types of medical imaging equipment may be different, and the integration of these data requires complex processing.

### 4.3. Summary

Robot-guided minimally invasive puncture based on a medical image has different advantages and disadvantages, as shown in Table 4. Ultrasound-guided robot puncture has good real-time performance, but the penetration ability of ultrasound is limited. For tissues and organs with large depth or complex structure, it is sometimes difficult to obtain accurate images, and the imaging effect is poor, and the overall puncture accuracy is not as good as that of CT/MRI. There is a certain amount of radiation in CT imaging, and CT/MRI equipment is large. The puncture robot guided by CT/MRI equipment is usually fixed next to the CT/MRI bed, which is complicated to disassemble and has some problems in adaptability. During the percutaneous puncture of the chest and abdomen, breathing movement will inevitably affect the accuracy of the robot puncture to some extent. On the one hand, respiratory movement caused the drift of puncture target; on the other hand, the quality of CT, MRI and other images decreased due to respiratory movement. The robot puncture guided by multimodal imaging technology has a good effect, but its technical route is relatively complicated.

## 5. Optimization Method of Medical Image Navigation Technology in Puncture Robot

The application of medical image navigation technology in puncture robots can improve surgical precision and safety, but there are some problems, including the following: (1) image accuracy problems: medical image navigation technology needs to be based on high-quality medical image data for navigation, but the image quality of some patients may not be good enough, or there may be problems such as artefacts, which may affect the puncture accuracy of the robot; (2) puncture accuracy problems caused by respiratory motion: Respiratory motion affects the accuracy of the image, while respiratory motion can also cause displacement of the puncture target. The application of medical image navigation technology in puncture robots still faces challenges and problems that require continuous improvement and optimization. The optimization methods of medical image navigation technology in puncture robots include the following.

### 5.1. Image Quality Optimization

Image quality optimization is very important for robot puncture. Liu et al. [96] improved the accuracy of lung nodule detection by computerized detection and diagnostic analysis of lung cancer to generate high-resolution CT images, which is of great value for diagnosing early lung cancer. Jiang et al. [97] present a motion-robust method for high-resolution 3D pulmonary MRI for robust motion-compensated image reconstruction. The overall image quality is improved compared to conventional reconstruction.

Different image data types can provide more comprehensive and accurate information [26]. Bi et al. [98] designed a robotic assist system for prostate intervention to improve the accuracy and real-time puncture. This robot can perform prostate biopsies under the guidance of MR (magnetic resonance) and transrectal ultrasound (TRUS) fusion imaging. In a laboratory setting, the final actual needle deviation from the desired target point, measured on a biomimetic tissue model, is less than 2.5 mm. Li et al. [99] used non-rigid software to fuse ultrasound and MRI models, and the robot performed a puncture biopsy of the prostate under the stereotactic guidance of the fused model. The experimental results showed that using fused images for puncture prostate biopsy is reliable and has a high lesion-detection rate, and the associated complications are significantly reduced.

High-resolution images can more clearly display the target tissue and surrounding structure, thus improving the accuracy of relevant disease detection, and improving the accuracy of puncture with good real-time performance, and reducing the occurrence of related complications.

### 5.2. Optimization of Puncture under Respiratory Motion

Respiratory motion causes the displacement of organs and organisational structures within the patient’s body. This movement may make it difficult for the robot to accurately hit the puncture target during puncture, thus affecting the success and safety of the procedure. The second, respiratory motion may cause changes in the shape and location of the target area in real-time imaging (e.g., CT, MRI, or ultrasound), making image guidance difficult. In addition, this variation may cause a delay in puncture due to the cyclic nature of respiratory movements.

Yan et al. [100] proposed an uncalibrated robotic puncture method based on respiratory motion. This method introduces an efficient approach for the online estimation of the Jacobian matrix using the Square Root Unscented Kalman Filter. This approach reduces computational time and enables dynamic tracking. Zhou et al. [101] proposed a CT-guided percutaneous lung nodule biopsy technique, which utilizes a respiratory motion model of the patient’s pulmonary nodules to plan the optimal puncture path and timing. The robot tracks and triggers puncture based on the respiratory phase. The puncture motion of the robot is completed within 0.4 s (It is a typical respiratory phase). The puncture accuracy is around 0.5 mm, and the standard deviation on non-resistant paths is approximately 0.1 mm. Lin et al. [102] utilized four-dimensional computed tomography (4DCT) images and respiratory motion to dynamically visualize the puncture path of a biopsy needle and synchronize the respiratory signal and the rendered image with the actual breathing, which in turn adjusts the robot’s puncture strategy and guides the puncture needle to the patient’s lesion.

In the process of robot puncture, the puncture optimization under breathing motion is of great significance. Through the monitoring and optimization of respiratory movement, real-time tracking and positioning of the puncture site can be achieved, thereby increasing the accuracy of surgery, reducing the risk of surgery, and reducing possible complications. This can not only improve the patient’s treatment effectiveness and comfort, but also strengthen the doctor’s control and confidence in the surgical process.

### 5.3. Algorithm Optimization

In the field of minimally invasive puncture robots, algorithms play a key role in image quality, path planning, automatic control and so on [98,103,104,105]. These algorithms are primarily used to assist physicians in performing precise and complex operations to improve surgical safety and efficiency.

During the procedure, the puncture needle needs to enter the body within a tightly controlled range. Using multiple sensors and data-fusion algorithms, the position of the robot arm can be estimated in real time, ensuring that the puncture needle follows a predetermined trajectory. Jiang et al. [106] present a precise positioning method for a puncture robot based on a PSO-Optimized BP Neural Network Algorithm. This studies a particle swarm optimization (PSO) back propagation (BP) neural network algorithm to solve the inverse kinematics problem of a UR3 robot based on six degrees of freedom, overcoming some disadvantages of BP neural networks. Wang et al. [107] designed an automated medical tele-robotic system for percutaneous puncture surgery. The robot’s control system is based on an Iterative Learning Control (ILC) algorithm, a dual-channel synchronous control system. The puncture actuator controlling channel is in charge of automatically inserting a needle and compensating for the motion of the internal organs. Gao et al. [108] used a master–slave control strategy based on the differential motion increment of the Jacobi inverse matrix with error compensation in the use of ultrasound-guided dual-arm robotic puncture, and this master–slave control algorithm reduces the computation time required for puncture, meets the demand for image guidance, and improves the success rate of the puncture procedure. Li et al. [109] proposed an improved linear rotation automatic calibration algorithm to reduce errors caused by manual calibration and system noise, aiming at the problem that the calibration accuracy of surgical gaps affects the accuracy of surgical system. Zhang et al. [110]. proposed a vision-based TCP calibration algorithm for traditional robots with poor puncture accuracy stability and high operation dependency, which improves the accuracy and stability of puncture.

In the process of robotic puncture, the significance and importance of algorithm optimization are reflected in the improvement of surgical accuracy, efficiency and safety. By optimizing the algorithm, the target organization can be located and tracked more accurately, thus reducing errors and risks. In addition, algorithmic optimization helps to more intelligently adjust and control the robot’s movements, making it more smoothly and naturally adaptable to complex surgical environments and needs.

## 6. Conclusions and Outlook

### 6.1. Limitations of the Prior Art

Medical image-guided robots that perform minimally invasive puncture surgery are the subject of a cutting-edge technology field, but there are some limitations in their application:

1. Image real time: Most medical imaging technologies (e.g., MRI or CT) do not usually provide real-time updated images, which can lead to robots operating based on slightly outdated data.
2. Image resolution: Although existing medical imaging technologies are already quite high resolution, higher image resolution and contrast may be required for certain minimally invasive procedures, such as nerve or blood vessel punctures.
3. Alignment of images to actual operations: Ensuring the accuracy of alignment between robotic operations and medical images can be challenging. Natural movements of the body (e.g., breathing or heartbeat) can lead to deviations between the actual tissue position and the image.
4. Cost issues: High-quality medical imaging equipment and the robotics associated with it are expensive and may be beyond the reach of some healthcare facilities.
5. Hardware and software compatibility issues: Medical imaging equipment and robotics from different manufacturers may have compatibility issues.
6. Safety and accuracy concerns: Although medical image guidance provides enhanced navigation, there is still a risk of misdirection leading to the mispicking of instruments.

Although medical image-guided robot puncture has made some progress, the existing technology still limits the accuracy of robot puncture. There is still a long way to go to solve the problems of real-time image, image resolution, image registration with actual operation, cost, hardware and software compatibility and other problems in the future, and relevant technologies still need to be researched and innovated.

### 6.2. Summary of Research Results

In recent years, significant results have been achieved in the research of medical image-guided robots for minimally invasive puncture procedures. These techniques mainly utilize computer-aided diagnosis, image processing, navigation and positioning, and robotics to control the puncture procedure precisely, mainly in the following aspects: (1) high-precision navigation: by combining medical images such as MRI, CT, and ultrasound with the robot system, real-time navigation and positioning can be achieved to improve the accuracy of puncture surgery; (2) high integration: researchers have successfully developed an integrated medical image guidance robot system that highly integrates the robot arm, image-processing software, navigation system, and puncture equipment; (3) human–machine collaboration: the robot system can achieve fine-tuning of the puncture needle during puncture surgery based on real-time image information in close collaboration with the physician; (4) adaptive adjustment: the robot system can work closely with the physician based on real-time image information; (5) human–machine collaboration: During the puncture procedure, the robotic system can work closely with the physician to achieve fine-tuning of the puncture needle based on real-time image information; (6) adaptive adjustment: through real-time analysis of the image data generated during the procedure, the robotic system can automatically adjust the puncture path and speed to cope with uncertainties such as tissue movement and deformation.

### 6.3. Looking Ahead to Future Research Directions

Although medical image-guided robot puncture has had more application results, there are still some challenges in some fields:

1. Real-time image acquisition and processing: In order to reduce image latency, research will focus on how to acquire and process medical images more quickly. With the help of advanced computing hardware and optimized algorithms, we can expect that in the near future, real-time or near-real-time high-quality medical images will be possible.
2. High-resolution and high-quality imaging technology: With advances in medical imaging technology, higher-resolution and higher-quality images can be expected in the future. This will not only provide more detailed information about human tissues, but also help robots to operate more accurately.
3. Application of deep learning in image processing: Deep learning has shown its powerful capabilities in many fields, including image recognition and processing. We can foresee that deep learning will play an increasingly important role in image processing, helping robots better understand image information and improve the accuracy and efficiency of operations.
4. Image alignment and tracking: Techniques to improve the alignment of images with actual surgical scenes are another important area of research. This may involve new alignment algorithms and may require new sensors or devices. In addition, how to track changes in human tissues in real time and how to adjust the operation of the robot according to these changes will also be part of the research on image alignment and tracking.
5. Multimodal image fusion: The use of different types of medical images can obtain more comprehensive information about the human body. How to effectively integrate this information so that the robot can understand the surgical environment more comprehensively and accurately will be an important research direction in the future.
6. Individual path planning: Each patient’s body structure is unique. Using medical image navigation technology, the puncture path can be customized for each patient, achieving truly individualized treatment. For some complex areas or diseases, traditional hand piercing may require a high level of skill and experience. With medical image navigation technology, the robot can automatically plan the optimal path, making the operation easier.

In general, the future research direction of the application of medical image navigation technology in minimally invasive puncture robots will focus on improving the speed, quality and resolution of image acquisition, improving image processing, registration and fusion technology, and providing patients with personalized puncture path planning to achieve more accurate and efficient minimally invasive surgery.

## Figures and Tables

**Table 1:** Application of medical imaging.

Applications	Example	Role and Advantages
Neurosurgery	Brain tumour removal, intracranial compression fracture repair, spinal surgery, etc.	Improved positioning accuracy for safer and more effective surgery [42,43]
Orthopedics	Joint replacement, fracture repositioning, spinal correction, etc.	Provides real-time 3D positioning information to reduce the risk of complications [44,45]
Otolaryngology	Sinus surgery, skull base surgery	Avoid damage to vital structures and improve the success rate of the procedure [46]
Oncology Treatment	Tumour resection surgery, radiation therapy	Accurately locate the tumour boundary, protect normal tissues, and improve surgical results and radiotherapy precision [47]
Cardiovascular Surgery	Coronary artery stenting, aortic valve replacement, etc.	Real-time display of blood vessel and organ locations for improved surgical safety and results [48,49]
Biological tissue biopsy	Biological tissue biopsy	Accurate localization of lesions, improved biopsy accuracy, and reduced risk of complication [50,51]

**Table 2:** Analysis of advantages and disadvantages of minimally invasive puncture robot.

**Advantage**	**Disadvantage**
High accuracy	High cost
Stable operation	High technical threshold
Easy training	Human–computer interaction problem
Strong replicability	
**Chance**	**Menace**
Personalized medicine solution	Regulatory restriction
Promote the development of high and new technologies	Technical failure risk
Interdisciplinary cooperation	Social acceptance
Global market expansion	Data security and privacy issues

**Table 3:** MRI-guided robotic puncture study tension.

Author	System Features	Operation Mode	Research Progress	Disadvantages
Fischer [87]	The system can position the needle for treatment by ejecting radioactive seeds or for diagnosis by collecting a tissue sample inside the magnet hole.	Semi-automatic	The overall accuracy of needle tip positioning was better than 0.25 mm and 0.5° relative to MR images.	The robot has limited space for operation, and the imaging effect will be affected when the motor is running.
Krieger [88]	Manipulator equipped with active reference tracking to encode the position of the needle path.	Automatic	Used in animal and clinical experiments	Lack of research on the effects of respiratory movements
Franco [89]	The robot is designed to operate inside a closed-hole MRI scanner	Semi-automatic	Save up to 30 min compared to manual MRI-guided laser ablation of liver tumours.	Lack of research on the effects of respiratory movements
Lim [90]	MRI safety robot made entirely of non-metallic parts with pneumatic actuators and optical encoders	Semi-automatic	The robot had no significant effect on the image quality of the MRI, with an average accuracy of 1.43 mm during puncture targeting.	Longer puncture time
Zhang [91]	Robot uses a control method that combines series–parallel robot forward kinematics with visual servo control.	Automatic	Repeat accuracy results showed that the average error for different lesions was 1.29~2.42 mm.	MRI image quality can be affected by the actuator

**Table 4:** Advantages and disadvantages of different medical imaging devices during puncture.

Type	Advantage	Disadvantage
US	Good real-time performance, no radiation, easy to move	The penetration ability is poor, and the overall imaging effect is not as good as CT and MRI
CT	Good imaging effect	The equipment is large in size, radiation when used, imaging time is long, easy to be affected by respiratory movement, and the use cost is high
MRI	Good imaging effect, no radiation	The equipment is large, the imaging time is long, and it is easily affected by respiratory movement and metal particles, and the use cost is high
Multimodal image	Integrated a variety of imaging technology, imaging quality is high.	The technology is complex and there are compatibility problems
